# Investigation of Gene Networks in Three Components of Immune System Provides Novel Insights into Immune Response Mechanisms against *Edwardsiella tarda* Infection in *Paralichthys olivaceus*

**DOI:** 10.3390/ani13152542

**Published:** 2023-08-07

**Authors:** Xiumei Liu, Xiaokai Bao, Zan Li, Quanqi Zhang

**Affiliations:** 1 College of Life Sciences, Yantai University, Yantai 264005, China; 2 School of Agriculture, Ludong University, Yantai 264025, China; 3 College of Marine Life Sciences, Ocean University of China, Qingdao 266003, China

**Keywords:** olive flounder, bacterial disease, infection, WGCNA, PPI network

## Abstract

**Simple Summary:**

*Paralichthys olivaceus*, a common marine fish, is susceptible to various harmful organisms. One such organism, *Edwardsiella tarda*, is a particularly potent pathogen that can cause severe illness and even death in fish during prolonged infections. The gills, blood, and kidneys, which are crucial components of the immune system in these fish, play vital roles in immune responses, including in the differentiation of immune cells, removal of diseased cells, and other immunity-related processes. In this study, we infected *Paralichthys olivaceus* with *Edwardsiella tarda* and analyzed the genetic information of three components at different timepoints after infection. By using advanced analytical techniques, we identified specific genes and gene networks associated with the immune response. Our innovative approach allowed us to gain a deeper understanding of how the immune system of *Paralichthys olivaceus* responds to *Edwardsiella tarda* infection in these important components. These findings provide valuable genetic resources for studying immunity in *Paralichthys olivaceus* and contribute to our knowledge of the molecular mechanisms underlying *Edwardsiella tarda* infections in fish.

**Abstract:**

As a quintessential marine teleost, *Paralichthys olivaceus* demonstrates vulnerability to a range of pathogens. Long-term infection with *Edwardsiella tarda* significantly inhibits fish growth and even induces death. Gills, blood, and kidneys, pivotal components of the immune system in teleosts, elicit vital regulatory roles in immune response processes including immune cell differentiation, diseased cell clearance, and other immunity-related mechanisms. This study entailed infecting *P. olivaceus* with *E. tarda* for 48 h and examining transcriptome data from the three components at 0, 8, and 48 h post-infection employing weighted gene co-expression network analysis (WGCNA) and protein–protein interaction (PPI) network analysis. Network analyses revealed a series of immune response processes after infection and identified multiple key modules and key, core, and hub genes including *xpo1*, *src*, *tlr13*, *stat1*, and *mefv*. By innovatively amalgamating WGCNA and PPI network methodologies, our investigation facilitated an in-depth examination of immune response mechanisms within three significant *P. olivaceus* components post-*E. tarda* infection. Our results provided valuable genetic resources for understanding immunity in *P. olivaceus* immune-related components and assisted us in further exploring the molecular mechanisms of *E. tarda* infection in teleosts.

## 1. Introduction

The Japanese flounder (*Paralichthys olivaceus*) constitutes a notable marine teleost species recognized for its tender meat and high nutritional value. This economically valuable fish is extensively cultivated along the coastal regions of China, Japan, Korea, and other nations [1,2,3]. In recent years, driven by mounting demand, aquaculture practices in these countries have intensified *P. olivaceus* farming densities. However, this surge in artificial breeding at elevated densities gravely impairs the surrounding aquatic environment and dramatically escalates the prevalence of bacterial, viral, and parasitic infections, consequently exerting detrimental effects on the growth and development of *P. olivaceus* populations [4,5]. Notably, bacterial infections severely compromise the vitality and overall health of *P. olivaceus*, often resulting in substantial mortality rates among the cultured fish [6,7].

*Edwardsiella tarda* is a Gram-negative bacterium that has been widely studied in various fish species [8]. Its highly infectious and viable characteristics allow it to penetrate the fish’s immune barrier and undergo rapid growth in vivo [9]. *E. tarda* infection has been documented in multiple fish species, including *Danio rerio* [10], *P. olivaceus* [11], and *Anguilla japonica* [12], where infection in *P. olivaceus* is associated with various symptoms such as pigment loss, eyeball protrusion, eye opacity, abdominal swelling, punctured bleeding of fins and skin, and abnormal swimming behavior. Consequently, *E. tarda* infection negatively impacts both the quality and quantity of cultivated *P. olivaceus*, resulting in profound economic losses within the aquaculture industry [13,14].

Fish immunity has drawn attention due to its complex responses and intricate molecular mechanisms [15,16]. As a focal area of research, fish immunology constitutes a considerable proportion of the broader aquatic science domain [17,18]. The gills, kidneys, and blood are important components in fish immunity [19]. Gills represent a prominent mucosal immune barrier in teleosts, regulating the expression of numerous immune-related genes [20], with the gill region serving as one of the primary exposure sites to pathogens, generating significant antibodies such as IgM and IgT post-pathogenic stimulation [21]. Kidneys, on the other hand, are the central immune and primary lymphoid tissues in teleost, secreting copious lymphocytes and macrophages [22]. Upon infection, they activate humoral and cellular immunity to resist pathogen invasion [23,24]. Simultaneously, prior studies have indicated that fish kidneys function analogously to mammalian bone marrow, promoting the production and maturation of erythrocytes and leukocytes [25]. Blood, the sole component of the immune system traversing an organism’s body, comprises a multitude of immune cells such as T lymphocytes, B lymphocytes, and neutrophils, which are responsible for hematogenic immunity [26,27,28]. In the event of pathogen invasion, blood exerts its immune function by transporting a large quantity of immune cells to various tissues, while phagocytes and leukocytes actively participate in blood immune processes by eliminating diseased cells [26,29]. Therefore, a comprehensive understanding of the immune function of these components following infection can yield invaluable insights into the immune response mechanisms of *P. olivaceus*.

The advent of high-throughput sequencing has revolutionized sequencing technologies, enabling faster and more accurate analysis of genetic information [30,31]. Among the methodologies used in high-throughput sequencing studies, weighted gene co-expression network analysis (WGCNA) plays a crucial role. By grouping genes into modules based on their expression patterns, WGCNA allows for the exploration of intricate relationships between genes and traits within these modules [32,33].

In this research, we applied WGCNA to investigate the co-expression networks in the three components of *P. olivaceus* during a 48 h *E. tarda* infection. Our aim was to gain insights into the associations between co-expressed genes and immune processes by performing Gene Ontology (GO) and Kyoto Encyclopedia of Genes and Genomes (KEGG) analyses. Additionally, we constructed protein–protein interaction (PPI) networks to examine the functional relationships among key genes across different modules. By identifying core and hub genes within these key modules, we aimed to elucidate the impact of bacterial infection on immune functions in the examined components. To validate the findings obtained through our WGCNA and PPI networks, we conducted quantitative RT-PCR (qRT-PCR) experiments. By comparing the expression patterns of core and hub genes determined by using the WGCNA and PPI networks with qRT-PCR results, we ensured the accuracy and reliability of our findings. These comprehensive analyses provide a valuable gene resource for understanding the immune response mechanisms of components of the immune system in *P. olivaceus* following *E. tarda* infection. Moreover, our research contributes to the broader field of immune-related studies in vertebrates.

## 2. Materials and Methods

### 2.1. Sample Acquisition

One-year-old *Paralichthys olivaceus* specimens were obtained from Yellow Sea Aquatic Product Co., Ltd. in Yantai, Shandong Province, China. The specimens were in good health, with an average length of 16.3 cm and weight of 70.5 g. To acclimate them to the new environment, we temporarily cultured the fish in a flow-through seawater system at 19 °C for one week. *Edwardsiella tarda* strain EIB202, isolated from diseased turbot, was acquired from the Key Laboratory of Microbial Oceanography, Ocean University of China. Prior to infection, *E. tarda* was cultured in the logarithmic phase at 28 °C in Luria-Bertani (LB) medium, centrifuged, and resuspended in a Ringer’s solution designed for marine teleosts with 2 × 10^7^ CFU/mL as the standard concentration.

This experiment was conducted in our laboratory. One hundred and thirty *P. olivaceus* with high vitality and no injuries were chosen and randomly divided into three groups: bacteria-challenge experiment group (BCEG), Ringer’s solution control group (RSCG), and blank control group (BCG). Sixty fish in BCEG received an intraperitoneal injection of 1 mL *E. tarda* suspension while 60 fish in RSCG were intraperitoneally injected with 1 mL Ringer’s solution. Ten fish in BCG were not injected with any bacteria or buffer solution. Blood, gill, and kidney samples were collected at 8 and 48 h post-infection. Likewise, samples from fish in BCG were collected at 0 h. Four fish were randomly selected at each timepoint for RNA extraction from the three components. Then, these four fish were mixed into two replicates. The remaining RNA was stored at −80 °C in preparation for quantitative RT-PCRs.

### 2.2. RNA-Seq and Screening of Differentially Expressed Genes

Libraries were constructed following the methods described in Li et al. [34]. Sequencing of the libraries was performed using the Illumina HiSeq 4000 platform. Clean reads were obtained by removing low-quality raw reads, reads with >10% unknown nucleotides, and those containing adaptor sequences. TopHat (version 2.0.13) was utilized for mapping clean reads to the reference genome of *P. olivaceus*. We employed Cufflinks (version 2.2.0) for transcript construction and abundance estimation from TopHat results, quantified as fragments per kilobase of transcript per million mapped reads (FPKM). Using Cuffdiff (version 2.2.0), we statistically compared differentially expressed genes (DEGs) between three groups of samples at the same timepoint in each component. The criteria for differential gene screening were *q*-value ≤ 0.05 and |log_2_ fold change| ≥ 1. We selected the union of DEGs at each timepoint of the three components as the gene dataset for constructing co-expression networks.

### 2.3. Construction of Gene Co-Expression Network

The construction of gene networks was performed using the R package WGCNA following the methodology outlined by Ponomarev et al. [35]. Initially, the DEGs were utilized as input for WGCNA, and the correlation coefficients between each gene pair were calculated. The determination of the optimal power parameter (β) was based on achieving a correlation between genes that adhered to the scale-free topology network, with the criterion of R-squared (R2) exceeding 0.8, as described by Zhang et al. [36]. Subsequently, a hierarchical clustering tree was constructed based on the calculated correlation coefficients. Genes demonstrating similar expression patterns were assigned to the same module while unassigned genes were grouped into the grey module. The thresholds for module clustering were set at a minimum module size of 25 and a cutting height of 0.86.

### 2.4. Identification of Key Modules and Genes

To identify the module most strongly associated with the target trait, we calculated correlation coefficients between modules and traits using module eigengenes. In this study, modules exhibiting the highest positive correlation with the trait were designated as key modules. To elucidate the principal functions of genes within these modules, we performed GO and KEGG enrichment analyses. Genes with high connectivity are more likely to hold significant biological importance. Consequently, we selected the top 20 most highly connected genes from each module as key genes, and among these, the three genes with the highest connectivity were designated as core genes. The interactions between key and core genes were visualized using Cytoscape. Furthermore, we investigated the functional linkages among genes in different modules by constructing protein–protein interaction networks utilizing STRING (version 11.5) [37]. Hub genes, which were identified based on their substantial number of protein interactions, were further validated by their higher connectivity.

### 2.5. qRT-PCR Validation

To evaluate the accuracy of the RNA-Seq results, we conducted quantitative reverse transcription-polymerase chain reactions (qRT-PCRs). The qRT-PCR protocol followed the methodology described by Wang et al. [38].

Total RNA was extracted using the TRIzol method based on the manufacturer’s instructions. RNA quality, concentration, and integrity were determined by using 1.2% agarose gel electrophoresis, NanoDrop 2000 spectrophotometer (Thermo Scientific, Waltham, MA, USA), and Agilent 2100 Bioanalyzer system (Agilent Technologies, Santa Clara, CA, USA), respectively. RNase-free DNase I was employed to eliminate DNA contamination. A volume of 350 μL of deproteinized solution (RW1) was added to the adsorption column (RA) and centrifuged at 12,000 rpm for 30 s, and the waste liquid was removed. Then, 50 μL DNase I was added to the solution and allowed to stand at room temperature for 15 min, followed by centrifugation at 12,000 rpm for 30 s after adding 350 μL RW1. Then, mRNA was purified using oligo (dT) magnetic beads from RNA and randomly sheared into pieces of approximately 200 base pairs in the fragmentation buffer. First-strand cDNA was synthesized from fragmented mRNAs under the action of reverse transcriptase and random hexamer primers. DNA polymerase I and RNase H was used to synthesize second-strand cDNA. After connecting the adapters and PolyA, PCR was used to isolate the adapter-modified fragments. We validated the expression levels of 21 core or hub genes, and their respective names and primer sequences can be found in Table 1. Gene-specific primers were designed using Primer Premier 5.0 software (PREMIER Biosoft, San Francisco, CA, USA). As a reference gene with stable expression, *β-actin* was employed. In each group, three biological replicates were used in qRT-PCR. We added 10 ng template cDNA to 20 μL SYBR Premix Ex Taq II (TaKaRa) solution. Then, cDNA was incubated for 5 min, undergoing 45 cycles of 95 °C for 15 s and 60 °C for 45 s in a LightCycler 480. Finally, the melting curve was analyzed, and fluorescent signal accumulation was recorded at the 60 °C, 45 s phase during each cycle by using a LightCycler 480 (Roche, Basel, Switzerland). Additionally, 2^−ΔΔCt^ method was used for statistical analysis.

## 3. Results

### 3.1. Screening of DEGs

**Figure 1:**
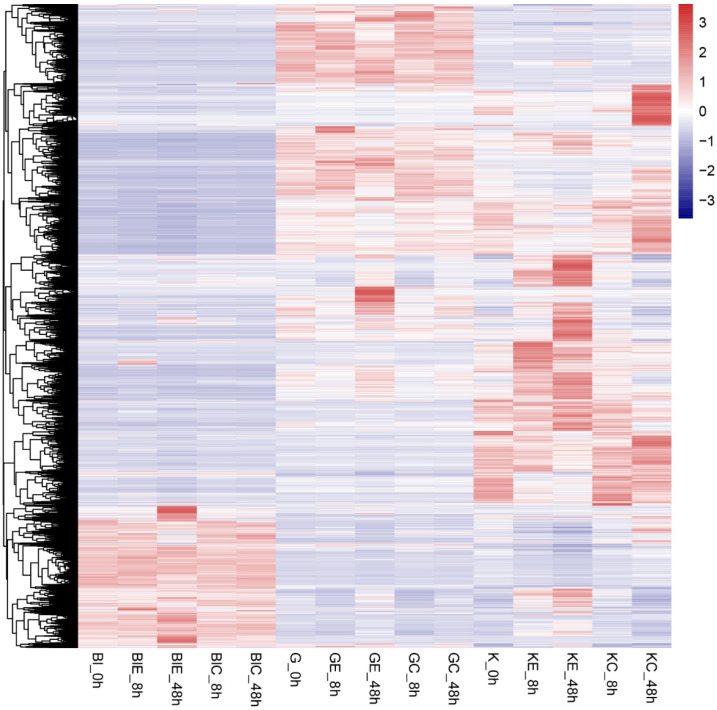
Hierarchical clustering heatmap of DEGs. Each column represents a group, and each row represents a gene. (Bl, G, and K stand for blood, gill, and kidney groups, BlE, GE, and KE represent infected blood, gill, and kidney groups, and BlC, GC, and KC indicate uninfected blood, gill, and kidney groups). Colors represent DEG expressions.

Following a comprehensive differential expression analysis, we identified 808 and 1265 DEGs in the blood of *P. olivaceus* infected with *E. tarda* at 8 and 48 h, respectively; 456 and 1037 DEGs in the gills at 8 and 48 h; and 1319 and 4439 DEGs in the kidneys at 8 and 48 h. Overall, we uncovered a total of 6459 DEGs (the union of all DEGs in the three components). These DEGs were subsequently employed for further analyses, and their distribution was visualized using a hierarchical clustering heatmap (Figure 1).

### 3.2. WGCNA

**Figure 2:**
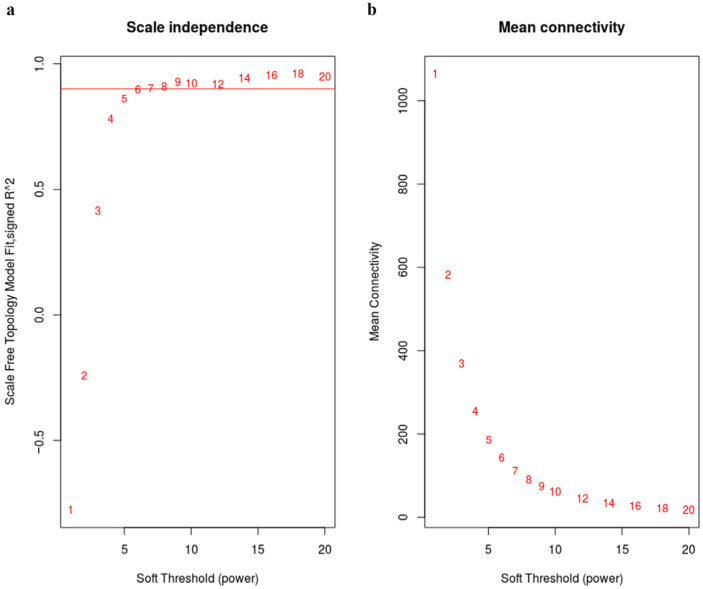
(**a**) The x-axis represents the power β and the y-axis stands for R2. The red line represents R2 = 0.8. (**b**) The x-axis indicates the power β and the y-axis represents mean connectivity.

**Figure 3:**
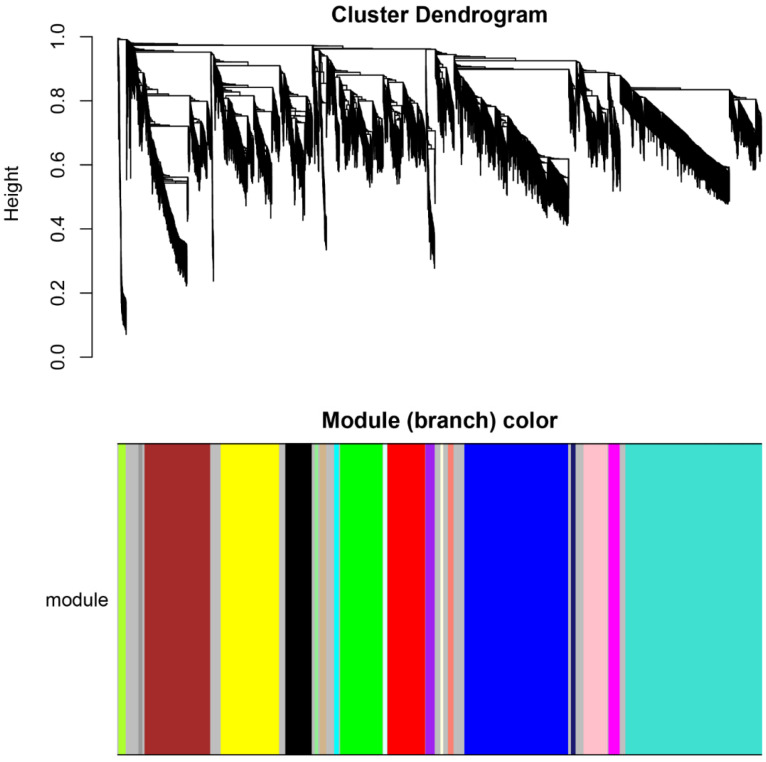
Hierarchical cluster tree of DEGs. The color strip indicates the module assignment, with the grey module encompassing unclustered DEGs. Each leaf node represents a DEG.

In this study, the 6459 DEGs were employed to construct gene co-expression networks. A scale-free topology model fit was constructed, and a power β value of 6 was selected to ensure that the network held high biological significance (Figure 2a). We concurrently assessed alterations in the mean connectivity of DEGs at different power β values (Figure 2b). Consequently, we identified twenty modules with module sizes ranging from 20 to 1359 (Table 2, Figure 3).

### 3.3. Identification and Functional Analysis of Key Modules

**Figure 4:**
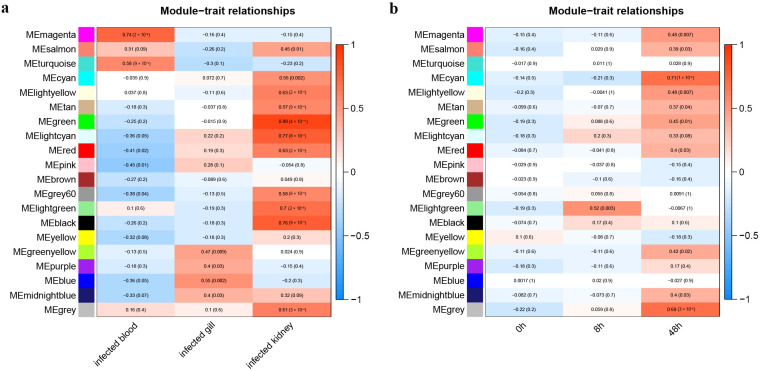
Heatmap display of module–trait relationships. (**a**) Correlation between modules and post-infection components. Each column corresponds to an infected component and each row stands for a module. The color scale from blue to red represents the range of correlation values, ranging from the maximum negative correlation to the maximum positive correlation. The values within each cell indicate the correlation coefficient and its associated *p*-value. (**b**) Correlation between modules and infection times.

**Figure 5:**
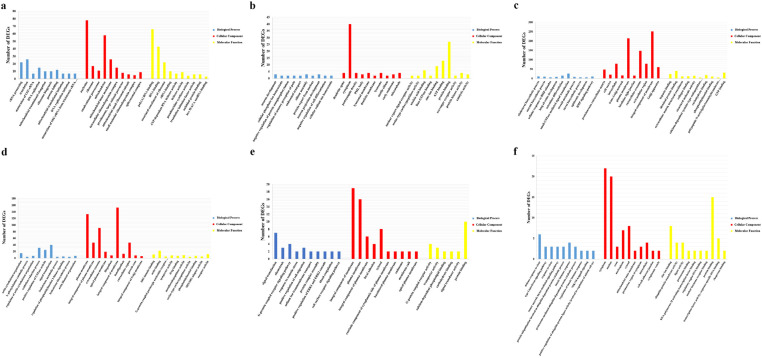
Level-3 GO terms of six modules. (**a**) GO terms enriched with DEGs in green module. The horizontal axis represents a GO term, the vertical axis stands for the number of DEGs enriched in terms, and the three colors indicate different GO term categories. (**b**) GO terms enriched with DEGs in magenta module. (**c**) GO terms enriched with DEGs in blue module. (**d**) GO terms enriched with DEGs in yellow module. (**e**) GO terms enriched with DEGs in lightgreen module. (**f**) GO terms enriched with DEGs in cyan module.

**Figure 6:**
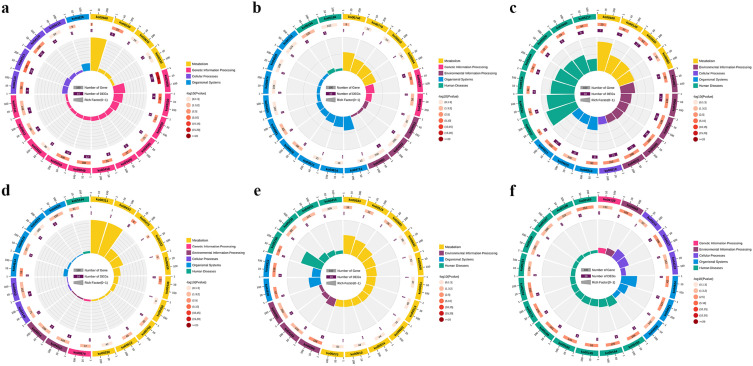
KEGG enrichment analysis results. (**a**) Top 20 level-2 KEGG pathways enriched in green module. The outermost circle represents the enriched level-2 KEGG pathways, and different colors stand for different classes; the second outer circle indicates the number of genes enriched into the pathway in the background gene set; the third circle represents the gene numbers enriched into the pathway in the input gene set; the Rich factor stands for the ratio of the number of genes in the input gene set enriched in the pathway to enriched gene numbers in the background gene set. (**b**) Top 20 level-2 KEGG pathways enriched in magenta module. (**c**) Top 20 level-2 KEGG pathways enriched in blue module. (**d**) Top 20 level-2 KEGG pathways enriched in yellow module. (**e**) Top 20 level-2 KEGG pathways enriched in lightgreen module. (**f**) Top 20 level-2 KEGG pathways enriched in cyan module.

Our investigation led to the identification of six modules exhibiting the strongest correlation with infection-responsive components or infection timepoints. These modules were based on gene eigenvalues. Specifically, the magenta, blue, and green modules demonstrated significant correlation with blood, gills, and kidneys post-infection, respectively (Figure 4a); DEGs in the yellow, lightgreen, and cyan modules played significant roles in components at 0, 8, and 48 h post-infection (Figure 4b). GO and KEGG enrichment analyses elucidated immune response mechanisms in the three components of *P. olivaceus* infected with *E. tarda*. For instance, 77 GO terms (covering biological processes, cellular components, and molecular functions) were enriched in the magenta module while 67, 281, 28, 492, and 149 terms were enriched in the cyan, green, lightgreen, yellow, and blue modules, respectively (Table S1, Figure 5). Several terms, such as protein export from nucleus, NIK/NF-κB signaling, innate immune response, and chemotaxis, were associated with immune responses. Similarly, pathways such as NOD-like receptor signaling, cAMP signaling, chemokine signaling, and the biosynthesis of antibiotics signaling in the six modules substantially contributed to immune response processes (Table S2, Figure 6).

### 3.4. Construction of PPI Network and WGCNA Key Networks

**Figure 7:**
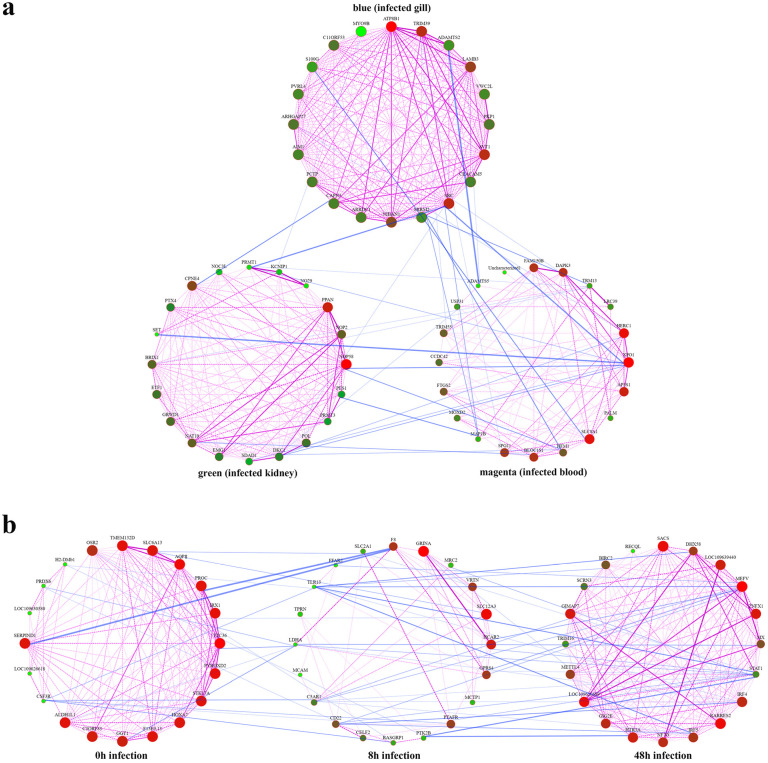
Gene interaction networks. (**a**) Gene networks representing different components. Each circle represents a gene, with the color and size indicating the level of gene connectivity. More red and larger circles represent higher connectivity. The lines between genes, varying in color and thickness within each module, indicate the strength of correlations between genes. Lighter and thicker lines represent higher correlation. Interactions between modules are shown by lines connecting genes. (**b**) Gene networks representing different infection timepoints.

**Figure 8:**
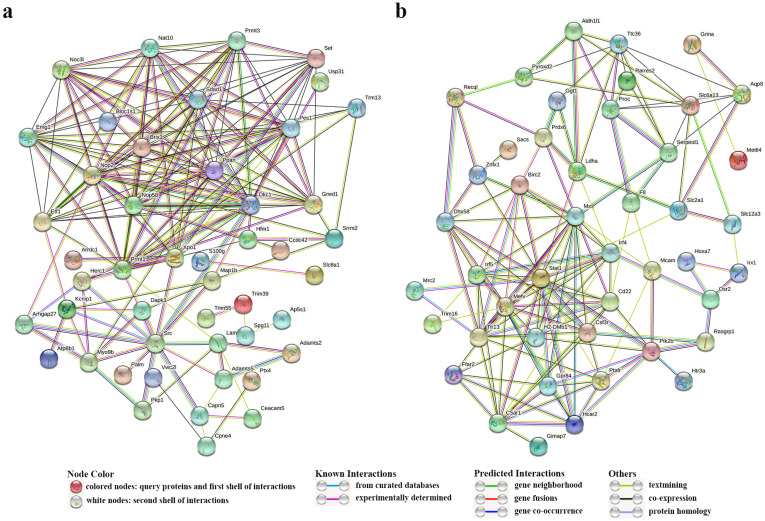
Protein–protein interaction networks. (**a**) Network illustrating immune interactions in the three infected components. Each circle represents a gene, and the connections between circles represent interactions between genes. (**b**) Network illustrating immune interactions within 48 h of infection.

We created distinct gene networks for the three modules demonstrating the highest correlation with infection-responsive components, employing 20 key genes from each module (Table S3, Figure 7a). The core genes exhibiting the highest connectivity in the magenta, green, and blue modules were *xpo1*, *herc1*, and *slc8a1*; *nop58*, *ppan*, and *cpne4*; and *atp8b1*, *src*, and *trim39*, respectively. These genes participated in immune responses of different components and played pivotal roles therein. Likewise, three networks correlating with infection timepoints were constructed (Table S4, Figure 7b). Core genes like *mefv*, *rarres2*, and *loc109626689* were identified as central to the cyan module, while *grina*, *slc12a3*, and *hcar2* emerged as core members of the lightgreen module. Moreover, *ttc36*, *aqp*, and *pyroxd2* were identified as core genes in the yellow module. These genes modulated immune responses at three distinct timepoints. Analyses of protein–protein interaction networks across modules revealed that *xpo1*, *src*, and *dkc1* (Figure 8a) might play crucial roles in the three infected components, and *stat*, *mefv*, and *tlr13* (Figure 8b) could have important effects within the first 48 h of infection. The interaction numbers and connectivity of hub genes are detailed in Table 3.

### 3.5. Validation of Hub Genes Using qRT-PCR

**Figure 9:**
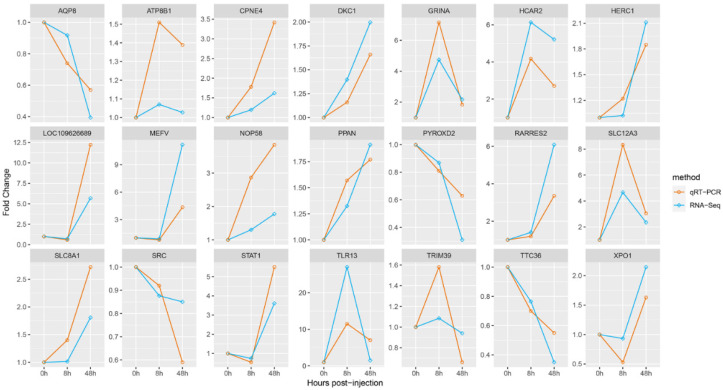
Comparison of gene expression between qRT-PCR and RNA-Seq results for core genes. The y-axis depicts fold change and the x-axis represents infection time.

To validate the accuracy of our transcriptome analysis results, we performed qRT-PCR experiments. The qRT-PCR data demonstrated concordance with the RNA-Seq findings, with all measured DEGs producing single products (Figure 9). The consistent gene expression trends observed using both RNA-Seq and qRT-PCR methods further support the reliability and accuracy of our RNA-Seq results.

## 4. Discussion

### 4.1. Comprehensive Analysis of WGCNA and PPI Network

The utilization of WGCNA and PPI networks constitutes two cutting-edge methodologies in the field of transcriptome analysis. These analytical techniques enable the determination of gene interactions and the identification of hub genes most closely associated with the subject matter being investigated. In previous research, these two methods have primarily been employed individually, rather than in conjunction with one another, to identify hub genes [38,39]. In the current study, we not only identified key and core genes with high connectivity within each key module but also innovatively ascertained numerous additional hub genes potentially involved in the regulation of *P. olivaceus* immunity through a combined approach using PPI network and WGCNA analysis. The results demonstrate that some genes possess both high connectivity and a considerable number of protein interactions, therefore designating them as hub genes likely implicated in the immune response processes in *P. olivaceus*. Concurrently, even though certain genes exhibit relatively low connectivity, their high number of protein interactions suggests a plausible significance in *P. olivaceus* immunity. The integrative analysis of these two methodologies allows for the discovery of numerous hub genes imperative for regulating the immune response of *P. olivaceus*, ultimately providing support for further studies examining the immune response processes in *P. olivaceus* following *E. tarda* infection.

### 4.2. Functional Analyses of Key Modules and Genes

We employed WGCNA analysis on 6459 DEGs to identify key genes affecting immune response processes in *P. olivaceus*, resulting in six modules with 120 key genes that were most relevant to specific components and infection timepoints. GO and KEGG analyses revealed numerous immune-related GO terms and KEGG signaling pathways enriched within various modules, signifying that module-enriched genes were intimately related to immune response processes. Among them, genes in magenta and lightgreen models were only enriched in one signaling pathway each. It was possible that there were too few genes in two modules to be enriched into multiple pathways. It was worth noting that the hub gene *xpo1,* enriched in the magenta model, was enriched in the only pathway. It involved and regulated biological immune responses processes, suggesting that *xpo1* might bear the main immune functions of fish blood. In our examination of the PPI network of 60 key genes in three key modules correlated with infection time and component data, we identified common interactions among these key genes even though they were associated with different infection times and component types. This observation implies that these genes may cooperatively regulate *P. olivaceus* immunity. By identifying six hub genes with high protein interaction numbers or high connectivity, we were able to posit that these hub genes play significant roles in the immune response processes of the selected components following *E. tarda* infection. Investigating these hub genes will aid in advancing our understanding of the immune response mechanisms in *P. olivaceus* after infection.

#### 4.2.1. Analysis of Magenta Module Associated with Blood Immunity

In this module, three genes, namely *xpo1*, *herc1*, and *slc8a1*, were identified as core regulators of immune response processes. Exportin 1 (*xpo1*), also referred to as chromosomal maintenance 1 (*crm1*), is a critical transport receptor belonging to the transport protein superfamily. It is ubiquitous in eukaryotic cells and mediates the transportation of numerous proteins and diverse RNA species [40]. Previous investigations have demonstrated that *xpo1* can facilitate the transport of several immune proteins, including PI3K, NF-κB, AKT, p53, and Wnt, to various tissues and organs [41,42]. These proteins significantly influence the growth, proliferation, apoptosis, inflammatory responses, and activation of T and B cells, among other processes [43]. Consequently, *xpo1* may indirectly modulate immune responses by regulating the transport of immune proteins. Moreover, *xpo1* impacts the transportation of growth-regulatory proteins. The upregulation of *xpo1* can enhance the transport of growth regulator proteins, thereby affecting immune cell proliferation [44]. In the current study, *xpo1* was identified as a core gene due to its high connectivity and interaction with six key genes in modules related to gill and kidney infection. We hypothesize that *xpo1* contributes to the growth and proliferation of immune cells within the blood and modulates *P. olivaceus* gill and kidney immune responses by transporting immune proteins and cells.

Hect domain and RCC1-like domain-containing protein 1 (*herc1*), a member of the HERC family proteins, regulates various cellular processes including cell cycle progression and cell signal transduction [45]. As a ubiquitin ligase, *herc1* primarily controls cell migration through the activation of the MKK and p38 modules [46]. Simultaneously, *herc1* modulates the RAF, MEK, and ERK signaling pathways, promoting the growth, proliferation, differentiation, and transport of immune cells [47].

The solute carrier family 8 member A1 (*slc8a1*) is an intracellular calcium regulator that mediates Na^+^ and Ca^2+^ exchange [48]. The upregulation of *slc8a1* suppresses the proliferation and migration of pathological and necrotic cells, promoting apoptosis [49]. Previous research has also demonstrated that *slc8a1* can regulate biological inflammatory responses [50]. While *slc8a1* has been extensively studied in humans, its function in fish remains largely unexplored. The immune roles of *slc8a1* in fish blood require additional investigation.

Collectively, these three core genes primarily function as transporters of immune cells and regulators of cell growth, differentiation, and apoptosis. The sustained upregulation of these genes suggests that immune response processes in *P. olivaceus* infected blood can effectively inhibit pathogen invasion.

#### 4.2.2. Analysis of Green Module Associated with Kidney Immunity

Three core genes primarily related to kidney immunity were found to potentially participate in and regulate various immune response processes. The majority of Box C/D non-coding RNA was located within nucleoli, wherein it interacted with several proteins to create RNA–protein complexes (RNPs). These complexes played a pivotal role in the modification and processing of pre-ribosomal RNA [51]. *nop58*, a primary interacting partner of Box C/D RNA, facilitated gene methylation upon binding, thus modulating the expression of immune-related genes [52,53].

*ppan*, a critical biogenic factor in nucleoli and mitochondria, regulated mitochondrial homeostasis [54]. Emerging research indicates that *ppan* is involved in immune response processes by interacting with the WNT signaling pathway, thus regulating the proliferation and differentiation of immune cells [55]. Furthermore, it contributes to the autophagy process responsible for degrading and recycling infected cells and organelles [56].

As a member of the copine family, *cpne4* primarily regulates Ca^2+^ concentrations in neurons [57]. Besides modulating calcium concentrations, *cpne4* notably participates in glycogen metabolism [58].

These three genes displayed the highest correlation with fish kidney immunity. Among them, *nop58* and *ppan* genes might be integral to immune response processes in *P. olivaceus* kidneys such as immune cell differentiation and immune-related signaling pathway regulation. Currently, no direct link between *cpne4* and immune response has been identified. However, we preliminarily hypothesize that *cpne4* may be associated with *P. olivaceus* kidney immunity. The specific functions of this gene require further exploration in future experiments.

#### 4.2.3. Analysis of Blue Module Associated with Gill Immunity

The gill plays a crucial role in initiating immune responses, specifically in the proliferation and differentiation of immune cells. In this study, we identified *atp8b1*, *src*, and *trim39* as potential regulators of gill immunity in *P. olivaceus*.

*atp8b1* is a phospholipid transporter that maintains epithelial cell stability via phospholipid transport [59]. Mutations in *atp8b1* have been associated with progressive familial intrahepatic cholestasis type 1 (*pfic1*) in humans and mice [60,61]. However, the function of *atp8b1* in fish has not been investigated, and its exact mechanisms remain unclear. We hypothesize that *atp8b1* in our study may regulate the production and apoptosis of gill epithelial immune cells through phospholipid transport, but further research is needed to confirm this hypothesis.

*src* is a non-receptor protein-tyrosine kinase that has been studied extensively in recent years [62]. It is implicated in regulating various cellular processes including proliferation, differentiation, and adhesion [63]. Notably, cell adhesion is closely associated with inflammatory responses and is a key step in immune responses [64]. *src* also participates in intracellular signaling that modulates the expression of multiple cytokines and immune complexes [65]. Previous research has demonstrated that *src* regulates the immune responses of immune receptors, C-type lectins, and integrins [65,66]. Salmond et al. [67] found that *src* influences the activation and differentiation of T cells by regulating the T-cell receptor signaling pathway, thereby modulating immune response processes. Our results indicate that *src* is enriched in the cell adhesion term, suggesting its significant immune function in the cell adhesion process in fish gills following *E. tarda* infection. Concurrently, *src* interacts with five genes enriched in other modules, suggesting its potential role in regulating the proliferation, differentiation, and other immune cellular processes in fish blood and kidneys.

The TRIM protein family plays an essential immunomodulatory role by activating various immune-related signaling pathways [67,68]. As a key immune gene in this family, *trim39* regulates the cell cycle and apoptosis of immune cells [68]. Previous studies have demonstrated that *trim39* is a significant regulator of innate immune responses in diverse organisms including humans and fish [68,69]. Moreover, *trim39* can activate the NF-κB signaling pathway, regulating cellular immunity, inflammatory response, and other crucial immune processes [70].

In conclusion, we posit that the gill, as the first line of defense against pathogen invasion, may play a significant role in *P. olivaceus* immunity following *E. tarda* infection. Investigating the functions of these three core genes could enhance our understanding of the immune response mechanisms in *P. olivaceus* gills.

#### 4.2.4. Analysis of Yellow Module Associated with 0 h Infection

*ttc36*, *aqp8*, and *pyroxd2* were identified as hub genes enriched in the yellow module, indicative of their potential role in the activation and proliferation of immune cells. *ttc36*, a member of the tetratricopeptide repeat (TPR) subfamily, has received little attention in previous research [71]. TPR proteins, recognized as scaffolds, have been implicated in numerous biological processes such as cell cycle regulation, apoptosis, protein transport and degradation, and resistance to bacterial infection [72]. In light of these previous findings, one might posit that *ttc36* similarly contributes to such functions. Notably, Jiang et al. demonstrated that *ttc36* could interact with HSP70 and upregulate its expression, leading to enhanced resistance against pathogen invasion [73]. This suggests a possible role for *ttc36* in the regulation of heat shock proteins and the modulation of biological immunity.

Aquaporin 8 (*aqp8*), a protein responsible for maintaining the intracellular water balance, has recently been implicated in processes such as tumorigenesis, transport, and apoptosis [74]. *aqp8* facilitates H_2_O_2_ transport and regulates various cellular processes [74,75]. Recent evidence has also revealed a role for *aqp8* in B cell signal transduction, wherein it modulates biological immunity through the activation and differentiation of B lymphocytes [76].

*pyroxd2*, localized within the inner membrane and matrix of mitochondria, is involved in the regulation of mitochondrial function [77,78]. Additionally, *pyroxd2* governs cellular processes, selectively promoting apoptosis in diseased cells while sparing their healthy counterparts [77].

These findings collectively suggest that certain *P. olivaceus* genes exhibit immune functions even in the absence of infection, perhaps as a defense mechanism against bacterial or viral contaminants at relatively low concentrations in seawater. Interestingly, these three core genes predominantly manifest in the kidney, with minimal expression in the blood. This observation implies that the kidney may serve as the primary organ for immune function in uninfected *P. olivaceus*, a hypothesis requiring substantiation through further experimentation.

#### 4.2.5. Analysis of Lightgreen Module Associated with 8 h Infection

The lightgreen module analysis identified three core genes, namely *grina*, *slc12a3*, and *hcar2*, which are associated with 8 h infection in *P. olivaceus*. *grina*, also referred to as *trim3*, is an anti-apoptotic protein present in cell membranes and has been implicated in the regulation of cell survival and apoptosis, as opposed to the regulation of Ca^2+^ concentration and neurotransmitter release [79]. Studies on *grina*-mediated apoptosis in various organisms such as mice, humans, and zebrafish have been conducted [79,80]. *grina* has been found to play a crucial role in regulating apoptosis during neuronal development and endoplasmic reticulum (ER) stress [81]. In light of these findings, we speculate that *grina* might regulate immune cell apoptosis in *P. olivaceus* following infection.

*slc12a3*, a sodium chloride cotransporter primarily expressed in the kidney, is regulated by multiple hormones such as aldosterone, glucocorticoids, and insulin [82,83]. The involvement of *slc12a3* in complex bio-ion concentration regulation processes implies its role in maintaining the ion balance within an organism [84,85]. Previous research has demonstrated *slc12a3*’s role in regulating Na^+^ concentrations in fish [86], which is further supported by our findings revealing an enrichment of the sodium transmembrane transport signaling pathway.

*hcar2* functions as a G-protein-coupled receptor involved in immune and inflammatory responses [34]. By regulating cAMP, NF-κB, and other signaling pathways, *hcar2* promotes T-cell activation and modulates cellular processes such as immune cell proliferation and differentiation, thereby influencing immune responses [34,87]. Our study posits that *hcar2* might play a similar role in *P. olivaceus* immunity.

All three core genes displayed a significant upregulation within the 8 h infection period, indicating that *P. olivaceus* may undergo intricate immune response processes following infection. It is noteworthy that while *hcar2*’s immune functions have not been reported previously, our results highlight a high expression of *hcar2* after *E. tarda* infection. Consequently, we propose that *hcar2* might have critical immune functions in fish, warranting further investigation in future studies.

#### 4.2.6. Analysis of Cyan Module Associated with 48 h Infection

In *P. olivaceus*, intricate immune responses emerged at 48 h post-infection, indicative of the potential involvement of three core genes as key regulatory factors in immune response processes. One such gene, *rarres2* (also known as chemerin), functions as an immunomodulatory factor primarily produced in adipose and cutaneous tissues [88]. Chemerin has been implicated in various immune-related processes, including microbial defense and inflammation, functioning as a leukocyte chemokine [89]. For instance, *rarres2* can recruit innate immune cells to inflammatory sites, subsequently eliciting inflammation [90]. Additionally, chemerin has been reported to directly modulate cellular processes such as the migration, invasion, and proliferation of pathological cells in humans [91]. Our findings suggest that *rarres2* may both be involved in the regulation of immune cell proliferation and differentiation as well as contribute to inflammatory processes.

Another core gene, *mefv*, has been implicated in regulating inflammatory responses and various cellular processes [92]. The pyrin protein encoded by *mefv* participates in innate immune processes, producing IL-1β to mediate inflammation [93]. Furthermore, *mefv* enrichment in the ubiquitin–protein transfer activity signaling pathway implies a potential role in the ubiquitination of immune proteins, thus regulating differentiation and apoptosis [92,93]. Despite extensive research on *mefv* in humans, its function in fish remains largely unexplored [94]. Consequently, the effects of differential *mefv* expression in fish warrant further investigation.

In this module, we also identified a core gene, *loc109626689*, which has yet to be annotated. Given its high connectivity, we postulate that *loc109626689* may be closely related to the immune response in fish. Functional analyses of other key and core genes in this module further suggest that *loc109626689* could participate in the regulation of inflammatory responses. Interestingly, the core genes exhibited elevated expression levels at 48 h post-infection and performed significant immune functions, excluding the uncertain gene *loc109626689*. To date, the immune functions of these genes have not been extensively examined in fish. We hypothesize that these genes may exert essential immune-related effects in *P. olivaceus* such as modulating inflammatory responses and diverse cellular processes.

### 4.3. Gene Function Analyses between Modules

In the present research, a novel and innovative approach was employed, combining PPI network analysis with the conventional WGCNA module connectivity analysis, to jointly pinpoint potential hub genes that could modulate the immune response in *P. olivaceus*. Our findings identified three crucial hub genes, namely *xpo1*, *dkc1*, and *src*, which appear to regulate immunity in *P. olivaceus*.

*dkc1* encodes the dyskerin ribonucleoprotein, implicated in promoting telomerase synthesis and maintaining telomere length and integrity, which in turn propels cell proliferation [95]. Concomitantly, it has been shown to regulate the proliferation, differentiation, senescence, and death of telomere-related cells [96]. Recent research has revealed the role of *dkc1* in controlling the proliferation, migration, and invasion of tumor cells [97]. Based on these findings, we tentatively posit that *dkc1* may regulate cellular processes such as the proliferation and migration of immune cells in *P. olivaceus*. *xpo1* and *src* were identified due to their higher protein interaction numbers and higher intramodular connectivity. Following our analysis, it is reasonable to suggest that these genes may exert a significant influence on the immune response processes in *P. olivaceus*.

*stat1*, *tlr13*, and *mefv* were pinpointed as regulators of the immune response processes in *P. olivaceus*, specifically within the first 48 h post-infection, based on interaction analyses. *stat1*, a key transcription factor affecting the JAK-STAT signaling pathway, modulates an array of cellular functions including proliferation, differentiation, migration, and apoptosis [70,98]. It has been implicated in various immune processes such as activating T cells, NK cells, and other immune cells and regulating innate immunity, adaptive immunity, and certain inflammatory responses [99]. Concurrently, it fosters the proliferation and activation of microglia to regulate neuroimmunity [100]. Toll-like receptors (TLRs) serve as pattern recognition receptors (PRRs) that are prevalent in a wide range of immune cells, participating in the regulation of immune response processes [101]. They have the capacity to recognize a broad spectrum of pathogens, encompassing bacteria, viruses, and parasites [102]. *tlr13*, a crucial member of the TLR family, plays an indispensable role in biological immune functionality. It has been shown to activate NF-κB, AP-1, type 1 interferon, and other immunoregulatory factors, thereby modulating innate immune responses [103]. In addition, it promotes the proliferation and differentiation of T cells, B cells, NK cells, neutrophils, and other immune cells, thus influencing biological immune response processes [101,104]. Remarkably, recent studies have demonstrated that *tlr13* is expressed in fish kidneys, potentially playing a pivotal role in fish kidney immunity [105]. Based on these conclusions, it is evident that these two hub genes perform essential functions in the immune response processes, leading us to speculate that they may regulate the proliferation and differentiation of immune cells, inflammatory response, the activation of immune factors, and other mechanisms in *P. olivaceus* infected with *E. tarda*. Moreover, *mefv*, another hub gene with higher protein interaction numbers, is speculated to partake in the inflammatory response to modulate immune response processes in *P. olivaceus*.

Our study indicates that the three component-associated hub genes were enriched in three distinct modules, signifying that the three components of *P. olivaceus’* immune system may exhibit comparable sensitivity to *E. tarda* and could all perform vital immune functions to resist and eliminate bacteria following infection. An additional three hub genes were identified in modules correlated with 8 and 48 h infection, potentially acting as hubs to participate in and regulate the immunity of *P. olivaceus* after *E. tarda* infection. The immune functions of these six genes within *P. olivaceus* remain ambiguous; therefore, further exploration of their roles will be valuable for understanding and characterizing the immune response processes in *P. olivaceus*.

## 5. Conclusions

In this study, we employed an innovative approach combining WGCNA and PPI network analysis to investigate the immune response mechanisms of *P. olivaceus* infected with *E. tarda*. Through comprehensive analyses, we identified six key functional modules. Within these modules, we identified nine core genes (*xpo1*, *herc1*, *slc8a1*, *nop58*, *ppan*, *cpne4*, *atp8b1*, *src*, and *trim39*) that were closely related to the immunity of the three examined components. Genes regulated unique immune response mechanisms in different components, indicating that fish component immunity may have a high specificity. Additionally, we discovered *mefv*, *rarres2*, *loc109626689*, *grina*, *slc12a3*, *hcar2*, *ttc36*, *aqp*, and *pyroxd2* as regulators of *P. olivaceus* immunity at different timepoints. The results indicated that *E. tarda* may inhibit ion transport, disrupt the ion balance, and promote cell apoptosis in *P. olivaceus* in the early stages of infection and induce severe inflammatory response during long-term infection.

Furthermore, our PPI network analysis, constructed by key genes in each group, identified six hub genes (*xpo1*, *dkc1*, *src*, *stat1*, *tlr13*, and *mefv*) that displayed high protein interaction numbers and connectivity. These hub genes are likely to have crucial immune functions in *P. olivaceus* when infected with *E. tarda*, regulating the proliferation and differentiation of immune cells and inducing inflammatory reactions to resist *E. tarda* infections. Notably, this research represents the first attempt to integrate PPI network analysis with WGCNA in order to identify multiple hub genes that potentially regulate the immunity of *P. olivaceus*.

The findings of our study lay the foundation for a deeper understanding of the immune response mechanisms in teleosts and hold promise for addressing the challenges of reduced production and disease in *P. olivaceus* under high-density artificial breeding conditions. By providing insights into the intricate interplay between genes and immune processes, our research contributes to the advancement of knowledge in the field of teleost immunity and has implications for the development of targeted strategies to enhance disease resistance in aquaculture settings.

## Figures and Tables

**Table 1:** List of primers used for qRT-PCR validation.

Gene Name	Forward Primer (5′-3′)	TM (°C)	Reverse Primer (5′-3′)	TM (°C)	Amplicon Length (bp)
*aqp8*	GTGTGCTTGGAGCTTCTATG	61	CAAACACTGCTCTTCCTACC	60	117
*atp8b1*	CCGACATCCTGTTGTTATCC	60	GGAGGCCCATCTTAAACTTC	60	102
*cpne4*	CACACACATCAGCCATCTAC	60	GTCCTGCTAATCCCTTCATAAC	60	119
*dkc1*	GATGTCTCATCGTGTGTGTAG	60	GAGTGTGTTCGCTCTCTATTG	60	117
*grina*	GCACAGACCCGTTATGATTT	61	AGCGATGTCGGAGTAATAGA	60	108
*hcar2*	CGTGACTCACACTGGATTT	60	GTGTCGGAGGAAGAATGAAG	60	125
*herc1*	CCAGTGTCTTGGTGGATTT	60	CGCATTGTCCCAGTCTTT	60	104
*loc109626689*	TATGGTGGAACAGCACAAAG	60	CGAAGCCCAGAGATGATAGA	61	101
*mefv*	TGGAGGAGGTGGAGTTTAT	60	CTCCTGCTCTGTTTGTACTC	60	125
*nop58*	CTTTCCCACTCCCTCTGTA	60	GAGTGTGGTGAACTGGTATG	60	123
*ppan*	CCACTCCTTCGTCTTTCATC	60	TCCTAACCTTCAGAGACTCG	60	110
*pyroxd2*	TGAACCAGACGGAGATAGG	60	CATCCAAGAGAGGCTGAATG	60	108
*rarres2*	TTCACTCGTTGGCACTTC	60	CTTGTCATCCAGCAGATTGT	60	107
*slc8a1*	CCCTCGATTGGTTGGTTATC	60	CTGCGTGATCGTCATCATAG	60	122
*slc12a3*	CGGGTTTCTACTTCCTCAAC	60	CTGCACAGACACTGAAAGAT	60	103
*src*	GAGTAAGCCCAAGGATTCAG	60	GGGACGGATGATAAGAGTTG	60	104
*stat1*	AGGAGTTGGAGCAGAAGT	60	GAGAGTTGGAGAGGAGGTT	60	108
*tlr13*	AAGGTGTTGGCGAGTAAAG	60	AGCCTTCTCGTCCGTATT	60	106
*trim39*	ACCAAGGTCGAACCAAAC	60	GAACCCGACTTGGCATTAT	60	107
*ttc36*	ACAGAGTCCAGACCTTAACC	61	CAGCATGGCAGTACAGTTAG	60	103
*xpo1*	GATTCCTCTGGGCTACATATTC	60	TCTCTGTCAGGCACTTCA	60	107

**Table 2:** List of module sizes.

Modules	DEG Numbers	Modules	DEG Numbers	Modules	DEG Numbers
magenta	111	lightcyan	40	yellow	585
salmon	55	red	378	greenyellow	72
turquoise	1359	pink	239	purple	94
cyan	46	brown	660	blue	1041
lightyellow	26	grey60	40	midnightblue	46
tan	66	lightgreen	34	grey	877
green	425	black	265		

**Table 3:** Summary of hub genes.

Gene Abbreviated Name	Gene Official Full Name	Protein Interaction Numbers	Connectivity
Infected components (blue, green, and magenta modules)			
*xpo1*	exportin 1	6	10.31
*dkc1*	dyskeratosis congenita 1, dyskerin	5	54.20
*src*	proto-oncogene tyrosine-protein kinase Src	5	16.78
Infection times (cyan, lightgreen, and yellow modules)			
*stat1*	signal transducer and activator of transcription 1	9	10.72
*tlr13*	toll-like receptor 13	8	1.31
*mefv*	MEFV innate immunity regulator, pyrin	6	16.95

## Data Availability

The Raw data has been uploaded in the Sequence Read Archive database on NCBI. The SRA accession numbers were SRR5713091, SRR5713092, SRR5713093, SRR5713094, SRR5713095, SRR5713096, SRR5713097, SRR5713098, SRR5713099, SRR5713100, SRR5713081, SRR5713082, SRR5713083, SRR5713084, SRR5713085, SRR5713086, SRR5713087, SRR5713088, SRR5713089, SRR5713090, SRR5713071, SRR5713072, SRR5713073, SRR5713074, SRR5713075, SRR5713076, SRR5713077, SRR5713078, SRR5713079, and SRR5713080.

## Supplementary Materials

The following supporting information can be downloaded at: https://www.mdpi.com/article/10.3390/ani13152542/s1, Table S1: GO terms in each module; Table S2: KEGG signaling pathways in each module; Table S3: Connectivity within modules and protein interaction numbers between modules in Figure 7a; Table S4: Connectivity within modules and protein interaction numbers between modules in Figure 7b.

## References

[1] Lou B., Gao L., Mao G., Shi H.L., Luo J.A. (2010). Analysis and evaluation of the nutritional components in the muscle of *Paralichthys olivaceus*. Acta Nutr. Sinica.

[2] Junhao N., Hu P., Li B., Jiang C. (2018). Nutritional comparison in muscle of wild, pond and factory cultured Japanese flounder (*Paralichthys olivaceus*) adults. Aquac. Res..

[3] Zhang H., Fu Y., Shi Z., Su Y., Zhang J. (2016). miR-17 is involved in Japanese Flounder (*Paralichthys olivaceus*) development by targeting the Cdc42 mRNA. Comp. Biochem. Physiol. Part B Biochem. Mol. Biol..

[4] Zhao X., Guan C., Dong D., Cui Y., Li J., Gao T. (2014). Comparison of Appearances and Nutritive Components of Muscles among Cage Farmed, Industrially Culrtured and Wild *Paralichthys olivaceus*. Period. Ocean. Univ. China.

[5] Zhang F., Qiu X., Liu Y., Wang J., Li X., Wang X. (2017). Expression analysis of three immune genes Interferon-gamma, Mx and Interferon regulatory factor-1 of Japanese flounder (*Paralichthys olivaceus*). Braz. Arch. Biol. Technol..

[6] Bin Park S., Nho S.W., Bin Jang H., Cha I.S., Lee J.-H., Aoki T., Jung T.S. (2017). Phenotypic and genotypic analysis of *Edwardsiella tarda* isolated from olive founder (*Paralichthys olivaceus*) and Japanese eel (*Anguilla japonica*). Aquaculture.

[7] Yan W., Qiao Y., He J., Wang Q., Chen Z., Ni F., Liu Y., Liu X., Zhang Q., Wang X. (2020). Characterisation, evolution and expression analysis of heat shock protein 20 genes from Japanese flounder (*Paralichthys olivaceus*) in response to *Edwardsiella tarda* infection. Aquaculture.

[8] Bin Park S., Aoki T., Jung T.S. (2012). Pathogenesis of and strategies for preventing *Edwardsiella tarda* infection in fish. Veter Res..

[9] Xu T., Zhang X.-H. (2014). *Edwardsiella tarda*: An intriguing problem in aquaculture. Aquaculture.

[10] Nikapitiya C., Chandrarathna H., Dananjaya S., De Zoysa M., Lee J. (2020). Isolation and characterization of phage (ETP-1) specific to multidrug resistant pathogenic *Edwardsiella tarda* and its in vivo biocontrol efficacy in zebrafish (Danio rerio). Biologicals.

[11] Yan W., Qiao Y., Qu J., Liu X., Zhang Q., Wang X. (2021). The hsp40 Gene Family in Japanese Flounder: Identification, Phylogenetic Relationships, Molecular Evolution Analysis, and Expression Patterns. Front. Mar. Sci..

[12] Hossain M., Kawai K., Oshima S. (2011). Immunogenicity of Pressure Inactivated *Edwardsiella tarda* Bacterin to *Anguilla japonica* (Japanese Eel). Pak. J. Biol. Sci..

[13] Wang L., Xiao J., Cui S., Wang Q., Wu H., Liu Q., Zhang Y. (2014). HU-induced polymorphous filamentation in fish pathogen *Edwardsiella tarda* leading to reduced invasion and virulence in zebrafish. Veter Microbiol..

[14] Wang J.-J., Sun L. (2015). *Edwardsiella tarda*-regulated proteins in Japanese flounder (*Paralichthys olivaceus*): Identification and evaluation of antibacterial potentials. J. Proteom..

[15] Plouffe D.A., Hanington P.C., Walsh J.G., Wilson E.C., Belosevic M. (2005). Comparison of select innate immune mechanisms of fish and mammals. Xenotransplantation.

[16] Zhu L.-Y., Nie L., Zhu G., Xiang L.-X., Shao J.-Z. (2013). Advances in research of fish immune-relevant genes: A comparative overview of innate and adaptive immunity in teleosts. Dev. Comp. Immunol..

[17] Xing J., Zhang Z., Luo K., Tang X., Sheng X., Zhan W. (2019). T and B lymphocytes immune responses in flounder (*Paralichthys olivaceus*) induced by two forms of outer membrane protein K from *Vibrio anguillarum*: Subunit vaccine and DNA vaccine. Mol. Immunol..

[18] Ye H., Lin Q., Luo H. (2018). Applications of transcriptomics and proteomics in understanding fish immunity. Fish Shellfish Immunol..

[19] Magrone T., Russo M.A., Jirillo E. (2019). Dietary Approaches to Attain Fish Health with Special Reference to their Immune System. Curr. Pharm. Des..

[20] Somamoto T., Miura Y., Nakanishi T., Nakao M. (2015). Local and systemic adaptive immune responses toward viral infection via gills in ginbuna crucian carp. Dev. Comp. Immunol..

[21] Li Z., Liu X., Cheng J., He Y., Wang X., Wang Z., Qi J., Yu H., Zhang Q. (2018). Transcriptome profiling provides gene resources for understanding gill immune responses in Japanese flounder (*Paralichthys olivaceus*) challenged with *Edwardsiella tarda*. Fish Shellfish. Immunol..

[22] Buchmann K. (2019). Immune response to Ichthyophthirius multifiliis and role of IgT. Parasite Immunol..

[23] Cha I.-S., Kwon J., Nho S.-W., Jang H.-B., Park S.-B., del Castillo C.S., Hikima J.-I., Aoki T., Jung T.-S. (2012). Kidney proteome responses in the teleost fish *Paralichthys olivaceus* indicate a putative immune response against Streptococcus parauberis. J. Proteom..

[24] Xu H., Xing J., Tang X., Sheng X., Zhan W. (2019). Immune response and protective effect against *Vibrio anguillarum* induced by DNA vaccine encoding Hsp33 protein. Microb. Pathog..

[25] Liu X., Li Z., Wu W., Liu Y., Liu J., He Y., Wang X., Wang Z., Qi J., Yu H. (2017). Sequencing-based network analysis provides a core set of gene resource for understanding kidney immune response against *Edwardsiella tarda* infection in Japanese flounder. Fish Shellfish. Immunol..

[26] Li Z., Liu X., Liu J., Zhang K., Yu H., He Y., Wang X., Qi J., Wang Z., Zhang Q. (2017). Transcriptome profiling based on protein–protein interaction networks provides a core set of genes for understanding blood immune response mechanisms against *Edwardsiella tarda* infection in Japanese flounder (*Paralichthys olivaceus*). Dev. Comp. Immunol..

[27] Ronald L., Alfaro A.C., Merien F., Burdass M., Venter L., Young T. (2019). In vitro immune response of chinook salmon (*Oncorhynchus tshawytscha*) peripheral blood mononuclear cells stimulated by bacterial lipopolysaccharide. Fish Shellfish. Immunol..

[28] Stosik M., Tokarz-Deptuła B., Deptuła J., Deptuła W. (2020). Immune Functions of Erythrocytes in Osteichthyes. Front. Immunol..

[29] Makesh M., Sudheesh P.S., Cain K.D. (2015). Systemic and mucosal immune response of rainbow trout to immunization with an attenuated Flavobacterium psychrophilum vaccine strain by different routes. Fish Shellfish Immunol..

[30] Cui W., Ma A. (2020). Transcriptome analysis provides insights into the effects of myo-inositol on the turbot *Scophthalmus maximus*. Fish Shellfish Immunol..

[31] Zhang J., Sun L. (2017). Transcriptome analysis reveals temperature-regulated antiviral response in turbot *Scophthalmus maximus*. Fish Shellfish Immunol..

[32] Cui W., Ma A., Huang Z., Wang X., Sun Z., Liu Z., Zhang W., Yang J., Zhang J., Qu J. (2020). Transcriptomic analysis reveals putative osmoregulation mechanisms in the kidney of euryhaline turbot *Scophthalmus maximus* responded to hypo-saline seawater. J. Oceanol. Limnol..

[33] Zhao L., Li Y., Lou J., Yang Z., Liao H., Fu Q., Guo Z., Lian S., Hu X., Bao Z. (2019). Transcriptomic Profiling Provides Insights into Inbreeding Depression in Yesso Scallop Patinopecten yessoensis. Mar. Biotechnol..

[34] Li M., Van Esch B.C.A.M., Wagenaar G.T.M., Garssen J., Folkerts G., Henricks P.A.J. (2018). Pro- and anti-inflammatory effects of short chain fatty acids on immune and endothelial cells. Eur. J. Pharmacol..

[35] Ponomarev I., Wang S., Zhang L., Harris R.A., Mayfield R.D. (2012). Gene Coexpression Networks in Human Brain Identify Epigenetic Modifications in Alcohol Dependence. J. Neurosci..

[36] Zhang B., Horvath S. (2005). A General Framework for Weighted Gene Co-Expression Network Analysis. Stat. Appl. Genet. Mol. Biol..

[37] Szklarczyk D., Franceschini A., Kuhn M., Simonovic M., Roth A., Minguez P., Doerks T., Stark M., Muller J., Bork P. (2010). The STRING database in 2011: Functional interaction networks of proteins, globally integrated and scored. Nucleic Acids Res..

[38] Wang Z., Wang Q., Wu H., Huang Z. (2021). Identification and characterization of amphibian SLC26A5 using RNA-Seq. BMC Genom..

[39] Zhang L., Hou R., Su H., Hu X., Wang S., Bao Z. (2012). Network Analysis of Oyster Transcriptome Revealed a Cascade of Cellular Responses during Recovery after Heat Shock. PLoS ONE.

[40] Azizian N.G., Li Y. (2020). XPO1-dependent nuclear export as a target for cancer therapy. J. Hematol. Oncol..

[41] Das A., Wei G., Parikh K., Liu D. (2015). Selective inhibitors of nuclear export (SINE) in hematological malignancies. Exp. Hematol. Oncol..

[42] Sun Q., Chen X., Zhou Q., Burstein E., Yang S., Jia D. (2016). Inhibiting cancer cell hallmark features through nuclear export inhibition. Signal Transduct. Target. Ther..

[43] Croft M. (2009). The role of TNF superfamily members in T-cell function and diseases. Nat. Rev. Immunol..

[44] Wang A.Y., Liu H. (2019). The past, present, and future of CRM1/XPO1 inhibitors. Stem Cell Investig..

[45] Schneider T., Martinez-Martinez A., Cubillos-Rojas M., Bartrons R., Ventura F., Rosa J.L. (2018). The E3 ubiquitin ligase HERC1 controls the ERK signaling pathway targeting C-RAF for degradation. Oncotarget.

[46] Pedrazza L., Schneider T., Bartrons R., Ventura F., Rosa J.L. (2020). The ubiquitin ligase HERC1 regulates cell migration via RAF-dependent regulation of MKK3/p38 signaling. Sci. Rep..

[47] Holloway A., Simmonds M., Azad A., Fox J.L., Storey A. (2014). Resistance to UV-induced apoptosis by β-HPV5 E6 involves targeting of activated BAK for proteolysis by recruitment of the HERC1 ubiquitin ligase. Int. J. Cancer.

[48] Rose C.R., Ziemens D., Verkhratsky A. (2019). On the special role of NCX in astrocytes: Translating Na^+^-transients into intracellular Ca^2+^ signals. Cell Calcium.

[49] He H., Wu S., Ai K., Xu R., Zhong Z., Wang Y., Zhang L., Zhao X., Zhu X. (2020). LncRNA ZNF503-AS1 acts as a tumor suppressor in bladder cancer by up-regulating Ca^2+^ concentration via transcription factor GATA6. Cell. Oncol..

[50] Shimizu C., Eleftherohorinou H., Wright V.J., Kim J., Alphonse M.P., Perry J.C., Cimaz R., Burgner D., Dahdah N., Hoang L.T. (2016). Genetic Variation in the SLC8A1 Calcium Signaling Pathway Is Associated with Susceptibility to Kawasaki Disease and Coronary Artery Abnormalities. Circ. Cardiovasc. Genet..

[51] Wang J., Huang R., Huang Y., Chen Y., Chen F. (2021). Overexpression of NOP58 as a Prognostic Marker in Hepatocellular Carcinoma: A TCGA Data-Based Analysis. Adv. Ther..

[52] Yang J., Zhang W., Sun J., Xi Z., Qiao Z., Zhang J., Wang Y., Ji Y., Feng W. (2017). Screening of potential genes contributing to the macrocycle drug resistance of *C. albicans* via microarray analysis. Mol. Med. Rep..

[53] Yang Z., Wang J., Huang L., Lilley D.M.J., Ye K. (2020). Functional organization of box C/D RNA-guided RNA methyltransferase. Nucleic Acids Res..

[54] Ruan W., Hu J., Zhou H., Li Y., Xu C., Luo Y., Chen T., Xu B., Yan F., Chen G. (2019). Intranasal wnt-3a alleviates neuronal apoptosis in early brain injury post subarachnoid hemorrhage via the regulation of wnt target PPAN mediated by the moonlighting role of aldolase C. Neurochem. Int..

[55] Dannheisig D.P., Beck E., Calzia E., Walther P., Behrends C., Pfister A.S. (2019). Loss of Peter Pan (PPAN) Affects Mitochondrial Homeostasis and Autophagic Flux. Cells.

[56] Pfister A.S., Keil M., Kühl M. (2015). The Wnt Target Protein Peter Pan Defines a Novel p53-independent Nucleolar Stress-Response Pathway. J. Biol. Chem..

[57] Liu X., Liu L., Wang J., Cui H., Chu H., Bi H., Zhao G., Wen J. (2020). Genome-Wide Association Study of Muscle Glycogen in Jingxing Yellow Chicken. Genes.

[58] Goel M., Li T., Badea T.C. (2019). Differential expression and sub-cellular localization of Copines in mouse retina. J. Comp. Neurol..

[59] Nayagam J.S., Williamson C., Joshi D., Thompson R.J. (2020). Review article: Liver disease in adults with variants in the cholestasis-related genes ABCB11, ABCB4 and ATP8B1. Aliment. Pharmacol. Ther..

[60] Deng L., Niu G.-M., Ren J., Ke C.-W., Loura L. (2020). Identification of ATP8B1 as a Tumor Suppressor Gene for Colorectal Cancer and Its Involvement in Phospholipid Homeostasis. BioMed Res. Int..

[61] Zarenezhad M., Dehghani S.M., Ejtehadi F., Fattahi M.R., Mortazavi M., Tabei S.M.B. (2019). In-silico Evaluation of Rare Codons and their Positions in the Structure of ATP8b1 Gene. J. Biomed. Phys. Eng..

[62] Roskoski R. (2004). Src protein–tyrosine kinase structure and regulation. Biochem. Biophys. Res. Commun..

[63] Levin V.A. (2004). Basis and importance of SRC as a target in cancer. Cancer Treat. Res..

[64] Lin H.-H., Hsiao C.-C., Pabst C., Hébert J., Schöneberg T., Hamann J. (2017). Adhesion GPCRs in regulating immune responses and inflammation. Adv. Immunol..

[65] Lowell C.A. (2010). Src-family and Syk Kinases in Activating and Inhibitory Pathways in Innate Immune Cells: Signaling Cross Talk. Cold Spring Harb. Perspect. Biol..

[66] De Kock L., Freson K. (2020). The (Patho)Biology of SRC Kinase in Platelets and Megakaryocytes. Medicina.

[67] Salmond R.J., Filby A., Qureshi I., Caserta S., Zamoyska R. (2009). T-cell receptor proximal signaling via the Src-family kinases, Lck and Fyn, influences T-cell activation, differentiation, and tolerance. Immunol. Rev..

[68] Shi Y., Hu S., Duan W., Ding T., Zhao Z. (2019). The distinct evolutionary properties of the tripartite motif-containing protein 39 in the Chinese softshell turtle based on its structural and functional characterization. Dev. Comp. Immunol..

[69] Wang W., Huang Y., Yu Y., Yang Y., Xu M., Chen X., Ni S., Qin Q., Huang X. (2016). Fish TRIM39 regulates cell cycle progression and exerts its antiviral function against iridovirus and nodavirus. Fish Shellfish Immunol..

[70] Zhang Y., Liu Z. (2017). STAT1 in cancer: Friend or foe?. Discov. Med..

[71] Hayden M.S., Ghosh S. (2008). Shared Principles in NF-κB Signaling. Cell.

[72] Song L., Guo X., Zhao F., Wang W., Zhao Z., Jin L., Wu C., Yao J., Ma Z. (2021). TTC36 inactivation induce malignant properties via Wnt-β-catenin pathway in gastric carcinoma. J. Cancer.

[73] Fesen K., Silveyra P., Fuentes N., Nicoleau M., Rivera L., Kitch D., Graff G.R., Siddaiah R. (2019). The role of microRNAs in chronic pseudomonas lung infection in Cystic fibrosis. Respir. Med..

[74] Ma J., Zhou C., Yang J., Ding X., Zhu Y., Chen X. (2016). Expression of AQP6 and AQP8 in epithelial ovarian tumor. Histochem. J..

[75] Prata C., Facchini C., Leoncini E., Lenzi M., Maraldi T., Angeloni C., Zambonin L., Hrelia S., Fiorentini D. (2018). Sulforaphane Modulates AQP8-Linked Redox Signalling in Leukemia Cells. Oxidative Med. Cell. Longev..

[76] Bertolotti M., Farinelli G., Galli M., Aiuti A., Sitia R. (2016). AQP8 transports NOX2-generated H2O2 across the plasma membrane to promote signaling in B cells. J. Leukoc. Biol..

[77] Liu H., Jiang X., Wang T., Yu F., Wang X., Chen J., Xie X., Fan H. (2017). Myeloid zinc finger 1 protein is a key transcription stimulating factor of PYROXD2 promoter. Oncol. Rep..

[78] Wang T., Xie X., Liu H., Chen F., Du J., Wang X., Jiang X., Yu F., Fan H. (2019). Pyridine nucleotide-disulphide oxidoreductase domain 2 (PYROXD2): Role in mitochondrial function. Mitochondrion.

[79] Chen K., Yang L.N., Lai C., Liu D., Zhu L.-Q. (2020). Role of Grina/Nmdara1 in the Central Nervous System Diseases. Curr. Neuropharmacol..

[80] Li L., Liu J.-C., Lai F.-N., Liu H.-Q., Zhang X.-F., Dyce P.W., Shen W., Chen H. (2016). Di (2-ethylhexyl) Phthalate Exposure Impairs Growth of Antral Follicle in Mice. PLoS ONE.

[81] Rojas-Rivera D., Armisén R., Colombo A., Martínez G., Eguiguren A.L., Díaz A., Kiviluoto S., Rodríguez D., Patron M., Rizzuto R. (2012). TMBIM3/GRINA is a novel unfolded protein response (UPR) target gene that controls apoptosis through the modulation of ER calcium homeostasis. Cell Death Differ..

[82] Cui H., Shan H., Miao M.Z., Jiang Z., Meng Y., Chen R., Zhang L., Liu Y. (2020). Identification of the key genes and pathways involved in the tumorigenesis and prognosis of kidney renal clear cell carcinoma. Sci. Rep..

[83] Urwin S., Willows J., Sayer J.A. (2019). The challenges of diagnosis and management of Gitelman syndrome. Clin. Endocrinol..

[84] Hruba P., Krejcik Z., Stranecky V., Maluskova J., Slatinska J., Gueler F., Gwinner W., Bräsen J.H., Wohlfahrtova M., Parikova A. (2019). Molecular Patterns Discriminate Accommodation and Subclinical Antibody-mediated Rejection in Kidney Transplantation. Transplantation.

[85] Zhou H., Liang X., Qing Y., Meng B., Zhou J., Huang S., Lu S., Huang Z., Yang H., Ma Y. (2018). Complicated Gitelman syndrome and autoimmune thyroid disease: A case report with a new homozygous mutation in the SLC12A3 gene and literature review. BMC Endocr. Disord..

[86] Takabe S., Inokuchi M., Yamaguchi Y., Hyodo S. (2016). Distribution and dynamics of branchial ionocytes in houndshark reared in full-strength and diluted seawater environments. Comp. Biochem. Physiol. Part A Mol. Integr. Physiol..

[87] Cavalli G., Dinarello C.A. (2017). Suppression of inflammation and acquired immunity by IL-37. Immunol. Rev..

[88] Mattern A., Zellmann T., Beck-Sickinger A.G. (2014). Processing, signaling, and physiological function of chemerin. IUBMB Life.

[89] Rennier K.R., Shin W.J., Krug E., Virdi G.S., Pachynski R.K. (2020). Chemerin Reactivates PTEN and Suppresses PD-L1 in Tumor Cells via Modulation of a Novel CMKLR1-mediated Signaling Cascade. Clin. Cancer Res..

[90] Buechler C., Feder S., Haberl E.M., Aslanidis C. (2019). Chemerin Isoforms and Activity in Obesity. Int. J. Mol. Sci..

[91] Goralski K.B., Jackson A.E., McKeown B.T., Sinal C.J. (2019). More Than an Adipokine: The Complex Roles of Chemerin Signaling in Cancer. Int. J. Mol. Sci..

[92] Kimura T., Jain A., Choi S.W., Mandell M.A., Johansen T., Deretic V. (2017). TRIM-directed selective autophagy regulates immune activation. Autophagy.

[93] Alghamdi M. (2017). Familial Mediterranean fever, review of the literature. Clin. Rheumatol..

[94] Georgin-Lavialle S., Ducharme-Benard S., Sarrabay G., Savey L., Grateau G., Hentgen V. (2020). Systemic autoinflammatory diseases: Clinical state of the art. Best Pr. Res. Clin. Rheumatol..

[95] Gaysinskaya V., Stanley S.E., Adam S., Armanios M. (2020). Synonymous Mutation in DKC1 Causes Telomerase RNA Insufficiency Manifesting as Familial Pulmonary Fibrosis. Chest.

[96] Kan G., Wang Z., Sheng C., Yao C., Mao Y., Chen S. (2021). Inhibition of DKC1 induces telomere-related senescence and apoptosis in lung adenocarcinoma. J. Transl. Med..

[97] Miao F.-A., Chu K., Chen H.-R., Zhang M., Shi P.-C., Bai J., You Y.-P. (2019). Increased DKC1 expression in glioma and its significance in tumor cell proliferation, migration and invasion. Investig. New Drugs.

[98] Mizoguchi Y., Okada S. (2021). Inborn errors of STAT1 immunity. Curr. Opin. Immunol..

[99] Meissl K., Simonović N., Amenitsch L., Witalisz-Siepracka A., Klein K., Lassnig C., Puga A., Vogl C., Poelzl A., Bosmann M. (2020). STAT1 Isoforms Differentially Regulate NK Cell Maturation and Anti-tumor Activity. Front. Immunol..

[100] Butturini E., Boriero D., de Prati A.C., Mariotto S. (2019). STAT1 drives M1 microglia activation and neuroinflammation under hypoxia. Arch. Biochem. Biophys..

[101] Vijay K. (2018). Toll-like receptors in immunity and inflammatory diseases: Past, present, and future. Int. Immunopharmacol..

[102] Kawai T., Akira S. (2006). Antiviral Signaling Through Pattern Recognition Receptors. J. Biochem..

[103] Shi Z., Cai Z., Wen S., Chen C., Gendron C., Sanchez A., Patterson K., Fu S., Yang J., Wildman D. (2009). Transcriptional Regulation of the Novel Toll-like Receptor Tlr13. J. Biol. Chem..

[104] Shi Z., Cai Z., Sanchez A., Zhang T., Wen S., Wang J., Yang J., Fu S., Zhang D. (2011). A Novel Toll-like Receptor That Recognizes Vesicular Stomatitis Virus. J. Biol. Chem..

[105] Sudhagar A., El-Matbouli M., Kumar G. (2020). Identification and Expression Profiling of Toll-Like Receptors of Brown Trout (*Salmo trutta*) during Proliferative Kidney Disease. Int. J. Mol. Sci..

